# A stay of execution: ATF4 regulation and potential outcomes for the integrated stress response

**DOI:** 10.3389/fnmol.2023.1112253

**Published:** 2023-02-07

**Authors:** Graham Neill, Glenn R. Masson

**Affiliations:** Division of Cellular and Systems Medicine, School of Medicine, University of Dundee, Dundee, United Kingdom

**Keywords:** ATF4, ISR, dimerization, target genes, PTM, apoptosis, cell division, synaptic plasticity

## Abstract

ATF4 is a cellular stress induced bZIP transcription factor that is a hallmark effector of the integrated stress response. The integrated stress response is triggered by phosphorylation of the alpha subunit of the eukaryotic initiation factor 2 complex that can be carried out by the cellular stress responsive kinases; GCN2, PERK, PKR, and HRI. eIF2α phosphorylation downregulates mRNA translation initiation *en masse*, however ATF4 translation is upregulated. The integrated stress response can output two contradicting outcomes in cells; pro-survival or apoptosis. The mechanism for choice between these outcomes is unknown, however combinations of ATF4 heterodimerisation partners and post-translational modifications have been linked to this regulation. This semi-systematic review article covers ATF4 target genes, heterodimerisation partners and post-translational modifications. Together, this review aims to be a useful resource to elucidate the mechanisms controlling the effects of the integrated stress response. Additional putative roles of the ATF4 protein in cell division and synaptic plasticity are outlined.

## Introduction

Translation in eukaryotes is the highly regulated process of converting the coding sequence contained in mRNAs into polypeptides. The translation process is exceedingly complex and is conducted primarily by the 80S ribosome alongside numerous accessory and regulatory proteins. Eukaryotic initiation factors (eIFs) control the initiation stage of translation where mRNA and the start codon recognising eIF2 ternary complex (a heterotrimeric complex required for the initiation of translation) load onto the 40S small ribosomal subunit (Sonenberg and Hinnebusch, 2009; Hinnebusch, 2014). This is followed by the 40s small and 60S large ribosomal subunits combining and translation elongation commencing.

The eIF2 heterotrimeric complex required for translation initiation consists of three subunits [eIF2α (EIF2S1), eIF2β (EIF2S2) and eIF2γ (EIF2S3); Kimball, 1999], a guanosine nucleotide (either GDP or GTP) and, when formed as the eIF2 ternary complex (only when eIF2 is bound by GTP), a methionine bound initiator tRNA (tRNA_i_^Met^). The high affinity of tRNA_i_^Met^ for the eIF2 complex is dependent on the complex being loaded with GTP (Kapp and Lorsch, 2004). Following a successful round of translation initiation, eIF2 is released from the rest of the translational machinery bound to GDP, which requires recycling to GTP to facilitate another round of tRNA_i_^Met^ loading. Recycling is carried out by the guanine nucleotide exchange factor (GEF) eIF2B. First, eIF2B removes eIF5 from eIF2 which remains bound following dissociation from the ribosome (Jennings et al., 2013). eIF2B then facilitates the exchange of GDP for GTP on eIF2, allowing tRNA_i_^Met^ to bind eIF2 to reform the eIF2 ternary complex (Boesen et al., 2004).

**Figure 1:**
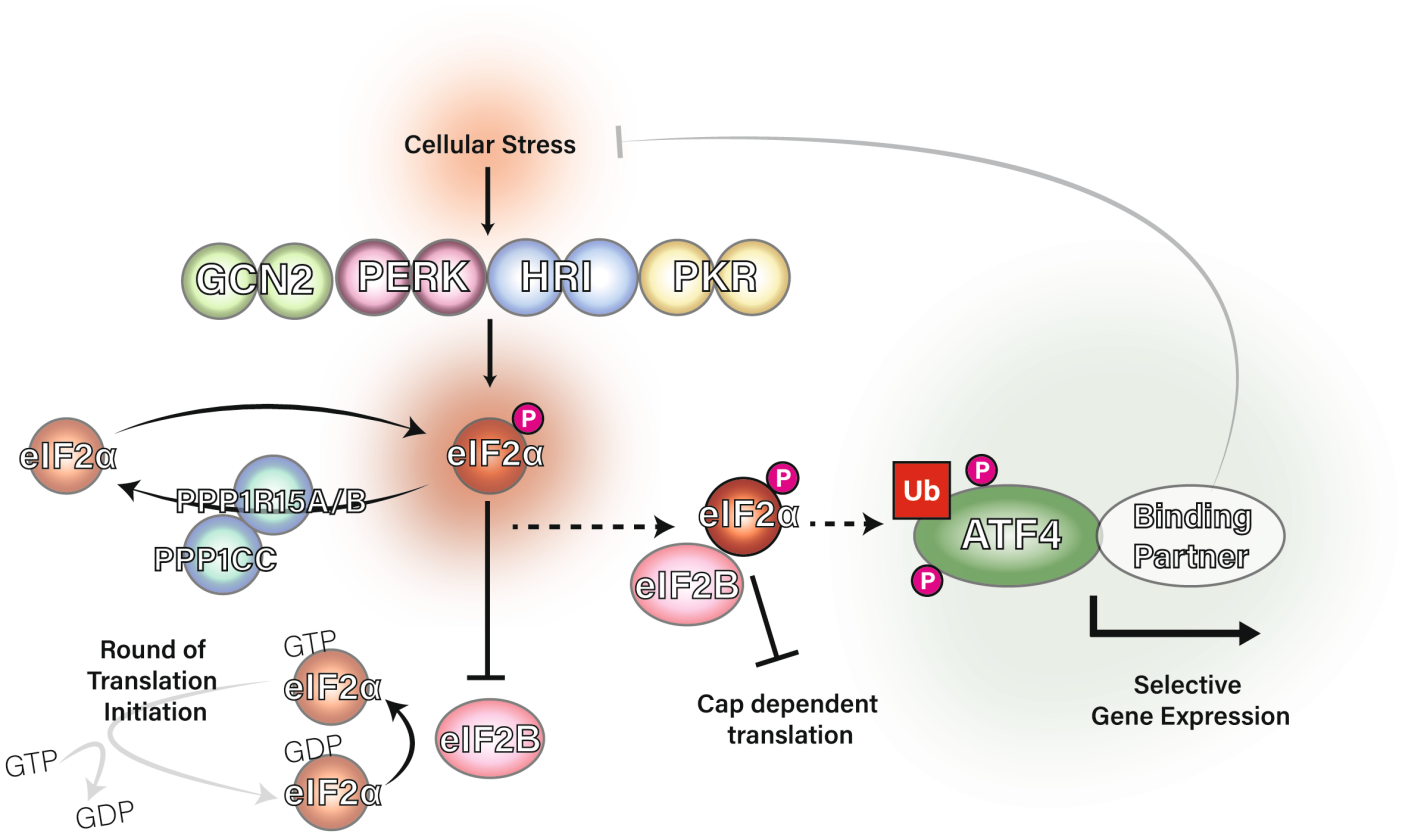
The integrated stress response and regulation of eIF2α. The eIF2 kinases GCN2, PERK, PKR and HRI phosphorylate eIF2α at serine 51 in response to a range of stress stimuli. DNAJC3 (also known as P58IPK) is known to inhibit the activity of GCN2, PERK and PKR (Roobol et al., 2015). Protein phosphatase 1 regulatory subunit 15A (PPP1R15A) and PPP1R15B are eIF2 adapters for the phosphatase catalytic subunit, PPP1CC, which downregulate the ISR *via* dephosphorylation of eIF2α at serine 51. PPP1R15B is constitutively expressed whereas PPP1R15A is induced by the integrated stress response in a negative feedback mechanism (Novoa et al., 2001; Brush et al., 2003; Jousse et al., 2003; Kojima et al., 2003). Phosphorylation of eIF2α causes eIF2 to inhibit eIF2B from acting as a guanine nucleotide exchange factor for eIF2 and the initiator Met-tRNAi cannot bind eIF2 unless GTP is present. Ultimately, eIF2α phosphorylation inhibits eIF2 ternary complex formation and therefore inhibits translation initiation leading to upregulation of ATF4.

The eIF2α subunit of eIF2 can be phosphorylated at serine 51 by one of four kinases: General Control Non-derepressible-2 (GCN2), Protein Kinase double-stranded RNA-dependent (PKR), PKR-like Endoplasmic Reticulum Kinase (PERK) or Heme-Regulated Inhibitor (HRI; Donnelly et al., 2013). These four kinases are distinctively regulated: GCN2 is typically activated by amino acid starvation, PERK by endoplasmic reticulum stress, PKR by viral infection and HRI by heme deficiency. Phosphorylation of eIF2α at serine 51 causes eIF2α to become a potent allosteric inhibitor of eIF2B through an alternative binding site (Bogorad et al., 2017; Adomavicius et al., 2019; Gordiyenko et al., 2019; Kashiwagi et al., 2019; Kenner et al., 2019). With eIF2B sequestered by eIF2α-P, it is unable to facilitate the guanine nucleotide exchange required for the creation of new ternary complexes. Reduction in eIF2 ternary complex formation is the trigger for the Integrated Stress Response (ISR). Figure 1 shows a graphical summary of eIF2 regulation in the ISR.

A hallmark of the ISR is the upregulation of the basic leucine zipper (bZIP) transcription factor Activating Transcription Factor 4 (ATF4; Harding et al., 2000; Wek, 2018). This is counterintuitive as one of the key features of the ISR is a global repression of translation initiation. However, mammalian ATF4 mRNA has an inhibitory upstream open reading frame (ORF) which overlaps onto the coding ORF for ATF4 (Vattem and Wek, 2004). When eIF2 ternary complex levels are high, the inhibitory upstream ORF in ATF4 mRNA is initiated in translation and the coding ORF is bypassed by the elongating ribosome. When eIF2 ternary complex concentrations are lower, the inhibitory upstream ORF can be bypassed, allowing translation initiation from the coding ORF for ATF4. This means that when eIF2α is phosphorylated and eIF2 ternary complex concentrations are reduced, ATF4 protein is upregulated. PPP1R15A (Lee et al., 2009; Young et al., 2015), ATF5 (Watatani et al., 2008; Zhou et al., 2008) and DDIT3 (Jousse, 2001; Palam et al., 2011; Young et al., 2016) are also upregulated by the ISR.

ATF4, previously known as CREB2, was initially described as a transcriptional repressor of the cAMP response element (CRE; Karpinski et al., 1992), however ATF4 can act as both an activator and inhibitor of transcription (Ameri and Harris, 2008). The exact selection of genes which are regulated by ATF4 is thought to be dependent on post-translational modifications and its binding partners, of which there are many. For example, ATF4 can heterodimerise with bZIP transcription factors JUN, FOS and FRA1 to bind cAMP response elements (CRE; Hai and Curran, 1991). ATF4 can also heterodimerise with CCAAT/enhancer-binding protein gamma (CEBPG) to bind CEBP–ATF response elements (CARE; Huggins et al., 2016). ATF4 can heterodimerise with DNA damage inducible transcript 3 (DDIT3; Su and Kilberg, 2008), also known as C/EBP homologous protein (CHOP) or GADD153. Increased expression of ATF4 and DDIT3, which are both upregulated by the ISR, can result in the induction of apoptosis (Gachon et al., 2001).

The ISR has been characterised to output two contrasting outcomes. If the ISR is activated at low levels or for a short amount of time, pro-survival pathways are upregulated; if the ISR is activated at high levels or for an extended amount of time, apoptotic pathways can be upregulated (Pakos-Zebrucka et al., 2016). The choice of ATF4 targeted gene expression has been attributed to combinations of ATF4 heterodimerisation partners (Pakos-Zebrucka et al., 2016), post-translational modifications (PTMs) and histone modifications surrounding target genes (Wortel et al., 2017).

The choice between survival and apoptosis under the ISR must be a regulated process and likely candidates involved in this regulation to be investigated in this review are categorised into three sections: ATF4-interacting proteins, PTMs and target genes. The aim of this review is to comprehensively search the literature available on ATF4 to provide a resource relevant to ATF4 regulation mechanisms.

## Methods

### Literature search

All searches were conducted up until the 15^th^ of November 2022.

For interaction studies, PubMed was searched using the following keywords: (ATF4 OR ATF-4 OR CREB2 OR CREB-2 OR ATF/CREB) AND (dimer OR heterodimer OR homodimer OR homodimeric OR heterodimeric OR heterodimerization OR two hybrid OR two-hybrid OR protein dimerization). Inclusion was allowed only for mammalian encoded ATF4. Further to this, the EMBL-EBI IntAct database (Orchard et al., 2014) was used with the search criteria ATF4 (P18848; *Homo sapiens* ATF4).

For post-translational modifications, three databases were searched; Uniprot (Bateman et al., 2021), PhosphoSitePlus (Hornbeck et al., 2015), and BioGRID (Oughtred et al., 2021) for ATF4/ATF-4.

For ATF4 target genes, PubMed was searched with the following keywords: (ATF4 OR CREB2 OR ATF/CREB) AND (ChIP OR chromatin immunoprecipitation). Only mammalian encoded ATF4 was allowed. For inclusion in the results table, the genes of interest required two independent observations. A complete list of all observed ATF4-interactors, target genes and PTMs can be found in Supplementary Tables 1–3, respectively.

### Information extraction

An author (GN) conducted initial searches and created a database of articles which met inclusion criteria. Two authors (GN and GRM) then read abstracts to determine whether they met the inclusion criteria. We extracted information on ATF4’s interaction partners, the organisms/cell lines the experiment (s) were conducted in, the methods employed, experimental design and statistical analysis. Special attention was given to publications using CREB2 to ensure antibodies used in older experiments were indeed against ATF4.

### Screening

Studies were included if they were (i) peer-reviewed primary research articles (i.e., Review articles were excluded), (ii) written in English, (iii) were conducted at the protein level with mammalian sequence ATF4. Only mammalian ATF4 was allowed (i.e., studies using yeast GCN4 or Sea Slug (Aplysia) ATF4 were excluded).

For interaction studies, there needed to be evidence at the protein level of a physical interaction between mammalian ATF4 and the binding proteins. This included methods such as yeast two hybrid, FRET and pull-downs.

For gene targets, we included genes that showed differential regulation with changes in ATF4 expression (evidenced or well-established methods of ATF4 induction) that showed evidence of *in vivo/ex vivo* binding of ATF4 to the gene promoter/enhancer using ChIP PCR, ChIP-Seq or reChIP/co-ChIP. Less robust and indirect evidence, such as knock-downs of ATF4 being correlated with gene target expression level changes alone, were not included if that target was only shown under those conditions.

For post-translational modifications, all observed human ATF4 post-translational modifications were allowed. Modifications that were inferred by species similarity alone were removed.

## Results

**Figure 2:**
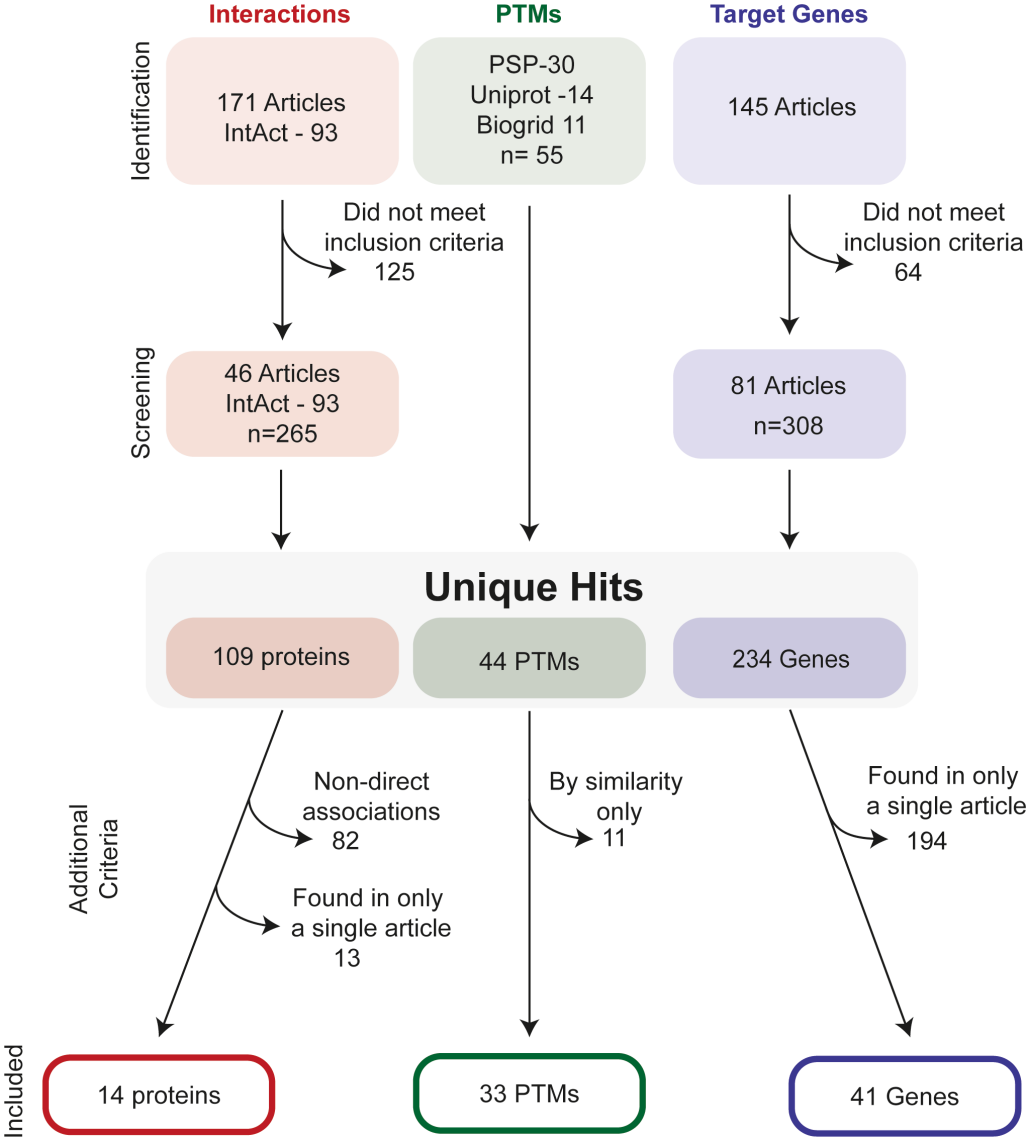
Selection process to identify relevant ATF4-interactors, PTMs and target-genes. For interactions studies, a total of 171 studies were investigated in addition to 93 interactions listed on the IntAct database, this was screened to identify an additional 65 unique interaction partners. After removal of interactions with non-direct evidence and those found only in a single publication, 14 proteins were identified. For PTMs, PhosphositePlus (PSP), Uniprot and Biogrid were all screened for PTMs. After checking for duplicates, 44 hits remained. Removal of predicted, i.e., as of yet observed PTMs in human, resulted in 33 PTMS. For target genes, 145 relevant articles were found from literature database searches, and after application of inclusion criteria, a total of 234 possible gene targets were identified. After stringency criteria were applied, a total of 41 hits were included.

**Figure 3:**
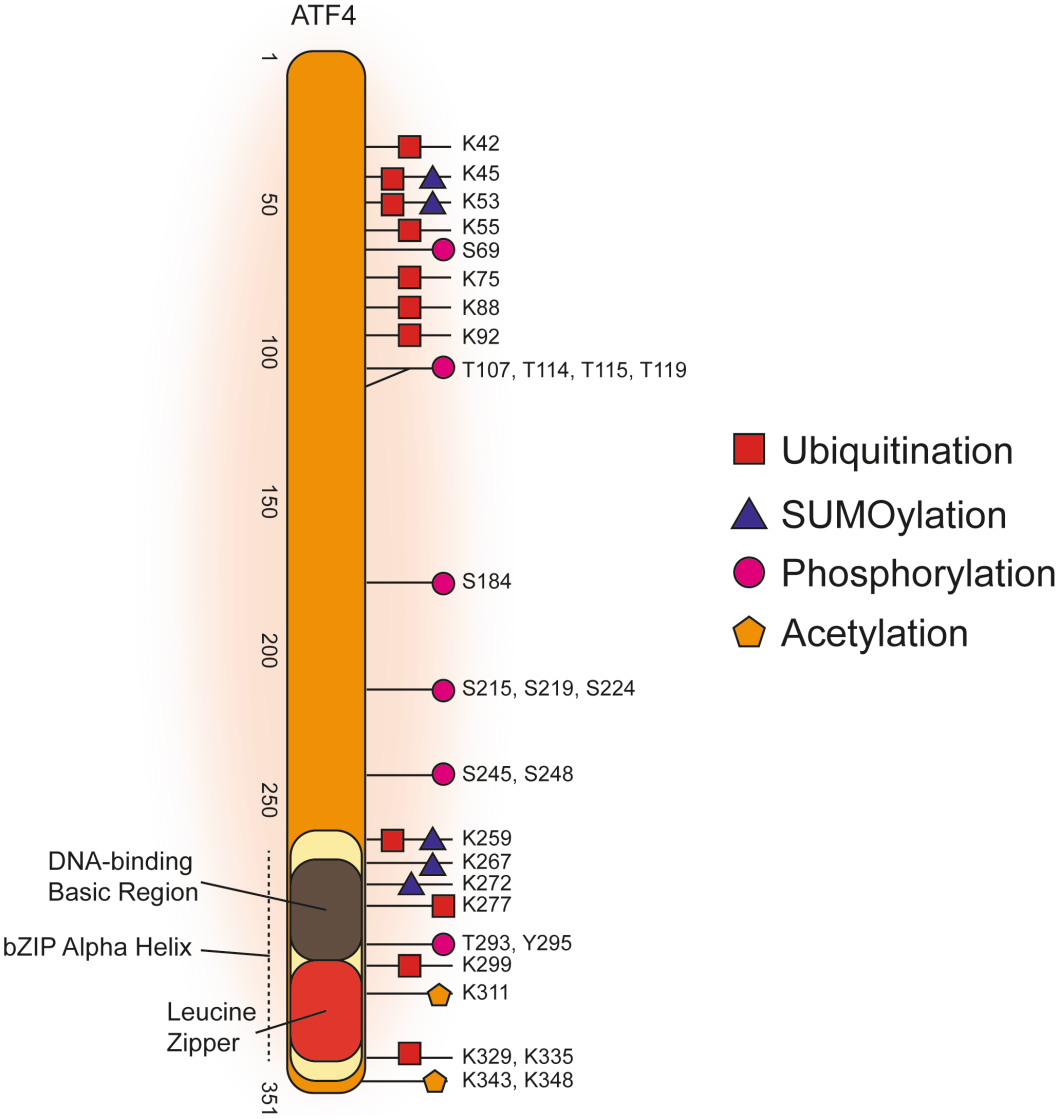
Post-translational Modifications of Human ATF4.

Figure 2 illustrates the process of how we searched for ATF4 PTMs, gene targets and interacting proteins. In total, we present evidence for 33 ATF4 PTMs (Figure 3), 14 ATF4 dimerisation partners (Table 1), and 41 genes that are regulated by ATF4 (Table 2).

**Table 1:** Direct ATF4 dimerisation partners.

ATF4-dimerisation partner	Description	References by species encoding ATF4
CEBPB	bZIP transcription factor	**Human**: Podust et al. (2001), Tominaga et al. (2008), Cohen et al. (2015). **Mouse**: Vallejo et al. (1993), Kawai et al. (1998), Mann et al. (2013), Ebert et al. (2020). **Unspecified**: Vinson et al. (1993), Lopez et al. (2007).
CEBPG	bZIP transcription factor	**Human**: Su and Kilberg (2008), Ravasi et al. (2010), Reinke et al. (2013), Rolland et al. (2014). **Mouse**: Huggins et al. (2016), Ebert et al. (2020), Luck et al. (2020). **Unspecified**: Vinson et al. (1993), Avitahl and Calame (1994).
JUN	bZIP transcription factor	**Human**: Bandyopadhyay et al. (2010), Reinke et al. (2013).**Porcine**: Kato et al. (1999). **Mouse**: Chevray and Nathans (1992), Fung et al. (2007). **Unspecified**: Hai and Curran (1991), Benbrook and Jones (1994), Steinmüller et al. (2001).
DDIT3/CHOP	bZIP transcription factor	**Human**: DeGrado-Warren et al. (2008), Su and Kilberg (2008), Reinke et al. (2013). **Rat**: Bromati et al. (2011). **Mouse**: Kawai et al. (1998), Gachon et al. (2001).
ATF4	bZIP transcription factor	**Human**: Su and Kilberg (2008), Reinke et al. (2013). **Mouse**: Vallejo et al. (1993), Mann et al. (2013), Ebert et al. (2020).
CEBPE	bZIP transcription factor	**Human**: Chih et al. (2004), Gombart et al. (2007), Reinke et al. (2013). **Mouse**: Chumakov et al. (2007). **Unspecified**: Vinson et al. (1993).
ATF3	bZIP transcription factor	**Human**: Su and Kilberg (2008), Wang et al. (2009), Reinke et al. (2013). **Mouse**: Kawai et al. (1998).
CEBPA	bZIP transcription factor	**Human**: Reinke et al. (2013). **Mouse**: Kawai et al. (1998), Ebert et al. (2020). **Unspecified**: Vinson et al. (1993).
NFE2L2/NRF2	bZIP transcription factor	**Human**: Su and Kilberg (2008), Reinke et al. (2013), Poh et al. (2020). **Rat**: He et al. (2001).
FOS	bZIP transcription factor	**Human**: Reinke et al. (2013). **Mouse**: Chevray and Nathans (1992). **Unspecified**: Hai and Curran (1991).
JUNB	bZIP transcription factor	**Human**: Wang et al. (2011b); Reinke et al. (2013). **Mouse**: Kawai et al. (1998).
CREBZF	bZIP transcription factor	**Human**: Reinke et al. (2013). **Unspecified**: Hogan et al. (2006).
MAF	bZIP transcription factor	**Human**: Reinke et al. (2013). **Mouse**: Ebert et al. (2020).
NFE2L1	bZIP transcription factor	**Human**: Reinke et al. (2013). **Mouse**: Murphy and Kolstø (2000).

**Table 2:** Targets known to be regulated by ATF4.

Product of ATF4 target gene	Brief description	Regulation	References by species
DDIT3/CHOP	bZIP transcription factor	Upregulation	**Human**: Bruhat et al. (2007), Shimizu et al. (2013), Wang et al. (2015), Lin et al. (2018), Bagheri-Yarmand et al. (2019), Örd et al. (2021). **Mouse**: Chérasse et al. (2007), Han et al. (2013), Farooq et al. (2022).
ASNS	Asparagine Synthetase	Upregulation	**Human**: Chen et al. (2004), Su and Kilberg (2008), Gjymishka et al. (2009), Burton et al. (2020), Örd et al. (2021). **Mouse**: Freundt et al. (2018).
TRIB3	Pseudokinase	Upregulation	**Human**: Su and Kilberg (2008), Carraro et al. (2010), Wang et al. (2015). **Rat**: Bromati et al. (2011). **Mouse**: Carraro et al. (2010), Han et al. (2013).
ATF3	bZIP transcription factor	Upregulation	**Human**: Pan et al. (2007), Lee et al. (2013). **Rat**: Zhou and Pan (2011). **Mouse**: Han et al. (2013), Sasaki et al. (2020).
VEGFA	Vascular Endothelial Growth Factor	Upregulation	**Human**: Su and Kilberg (2008), Wang et al. (2012), Kim et al. (2020). **Mouse**: Oskolkova et al. (2008), Freundt et al. (2018).
MTHFD2	Mitochondrial bifunctional dehydrogenase	Upregulation	**Human**: Wang et al. (2015), Örd et al. (2021). **Mouse**: Han et al. (2013), Freundt et al. (2018).
SLC7A11	Cystine/glutamate antiporter xCT	Upregulation	**Human**: Wang et al. (2015), Örd et al. (2021), Ferguson et al. (2022). **Mouse**: Han et al. (2013).
FGF21	Fibroblast growth factor	Upregulation	**Human**: Tao et al. (2022). **Mouse**: Örd et al. (2018), Sasaki et al. (2020).
AARS	Aminoacyl-tRNA synthetase	Upregulation	**Mouse**: Han et al. (2013), Shan et al. (2016), Freundt et al. (2018).
CEBPB	bZIP transcription factor	Upregulation	**Human**: Chen et al. (2005), Wang et al. (2015). **Mouse**: Guo et al. (2019).
CHAC1	Glutathione-specific gamma-glutamylcyclotransferase	Upregulation	**Human**: Crawford et al. (2015), Wang et al. (2015). **Mouse**: Juliana et al. (2018).
DDIT4/REDD1	Negative regulator of mTOR	Upregulation	**Human**: Wang et al. (2015), Örd et al. (2021), Han et al. (2021).
GPT2	Mitochondrial glutamic--pyruvic transaminase	Upregulation	**Human**: Wang et al. (2015). **Mouse**: Han et al. (2013), Juliana et al. (2018).
LC3B/ MAP1LC3B	Autophagy protein	Upregulation	**Human**: Shen et al. (2015), Zhong et al. (2022). **Rat**: Cai et al. (2022).
PPP1R15A /GADD34	Protein phosphatase 1 adaptor for eIF2	Upregulation	**Human**: Wang et al. (2015). **Mouse**: Han et al. (2013), Sasaki et al. (2020).
PSAT1	Phosphoserine aminotransferase	Upregulation	**Human**: Gao et al. (2017), Örd et al. (2021). **Mouse**: Freundt et al. (2018).
WARS	Aminoacyl-tRNA synthetase	Upregulation	**Human**: Wang et al. (2015). **Mouse**: Han et al. (2013), Shan et al. (2016).
ALDH18A1	Mitochondrial aldehyde dehydrogenase	Upregulation	**Mouse**: Han et al. (2013), Freundt et al. (2018).
ATG7	Autophagy related protein	Upregulation	**Human**: Zhong et al. (2022). **Rat**: Cai et al. (2022).
BGLAP (Osteocalcin)	Bone gamma-carboxyglutamic acid-containing protein	Upregulation	**Mouse**: Tominaga et al. (2008), Yu et al. (2009).
CDSN	Corneodesmosin	Upregulation	**Mouse**: Han et al. (2013), Sasaki et al. (2020).
EIF2S2	eIF2 subunit (eIF2β)	Upregulation	**Mouse**: Han et al. (2013), Freundt et al. (2018).
EPRS	Aminoacyl-tRNA synthetase	Upregulation	**Mouse**: Han et al. (2013), Shan et al. (2016).
FGF19	Fibroblast growth factor	Upregulation	**Human**: Shimizu et al. (2013), Lang et al. (2021).
GARS	Aminoacyl-tRNA synthetase	Upregulation	**Mouse**: Han et al. (2013), Shan et al. (2016).
GDF15	Growth/differentiation factor	Upregulation	**Human**: Wang et al. (2015), Li A. et al. (2021).
HERPUD1	Homocysteine-responsive ER-resident ubiquitin-like domain	Upregulation	**Human**: Wang et al. (2015). **Mouse**: Freundt et al. (2018).
HSPA5	Molecular chaperone	Upregulation	**Human**: Wang et al. (2015). **Mouse**: Han et al. (2013).
IARS	Aminoacyl-tRNA synthetase	Upregulation	**Mouse**: Han et al. (2013), Shan et al. (2016).
JDP2	bZIP transcription factor	Upregulation	**Human**: Wang et al. (2015). **Mouse**: Freundt et al. (2018).
KDM7A	Histone lysine demethylase	Upregulation	**Human**: Wang et al. (2015). **Mouse**: Han et al. (2013).
LARS	Aminoacyl-tRNA synthetase	Upregulation	**Mouse**: Han et al. (2013), Shan et al. (2016).
miR-552	Micro RNA 552	Upregulation	**Human**: Feng et al. (2022). **Mouse**: Feng et al. (2022).
MKNK2	MAP kinase-interacting serine/threonine-protein kinase	Upregulation	**Human:** Wang et al. (2015), Bagheri-Yarmand et al. (2019).
NARS	Aminoacyl-tRNA synthetase	Upregulation	**Mouse**: Han et al. (2013), Shan et al. (2016).
PTGS2/COX2	Prostaglandin-endoperoxide synthase	Upregulation	**Human**: Xiao et al. (2011), Di et al. (2018).
SARS	Aminoacyl-tRNA synthetase	Upregulation	**Mouse**: Han et al. (2013), Shan et al. (2016).
SQSTM1/P62	Autophagosome cargo protein	Upregulation	**Rat**: Cai et al. (2022). **Mouse**: Han et al. (2013).
VARS	Aminoacyl-tRNA synthetase	Upregulation	**Mouse**: Han et al. (2013), Shan et al. (2016).
VLDLR	Very-low-density-lipoprotein receptor	Upregulation	**Human**: Wang et al. (2015). **Mouse**: Han et al. (2013).
YARS	Aminoacyl-tRNA synthetase	Upregulation	**Mouse**: Han et al. (2013), Shan et al. (2016).

### ATF4-interaction proteins

Forty four interacting partners of ATF4 were identified in a targeted PubMed database search, with an additional 65 provided by the IntAct database (Orchard et al., 2014). In total, 109 ATF4-interacting proteins were identified (Supplementary Table 1), 41 (30%) of these contained a leucine zipper motif of which 33 are bZIP transcription factors. Twenty seven of the bZIP transcription factors were verified as direct ATF4-interacting heterodimerisation partners, with multiple references for 14 (Table 1). Direct interaction was mostly determined by an appreciable work (Reinke et al., 2013) that purified bZIP transcription factor proteins and measured their dimerisation with Förster resonance energy transfer (FRET). CEBPB was confirmed as an ATF4 dimerisation partner based on X-ray crystallography of their bZIP domains (Podust et al., 2001). Five ATF4-interacting basic helix–loop–helix transcription factors were found as well as two transcriptional co-activators, CREBBP and TRIM24. Eight ATF4-interacting proteins were characterised as being involved with cellular division or the cytoskeleton. Six ATF4-interacting proteins were found to be integral to neural synapsis; GABBR1, GABBR2, DISC1, SNAP29, NLGN3, and APH1A. Four proteins involved in ubiquitination were identified; βTRCP, ABRO1, ASB7, and MDM2. Pro-apoptotic proteins, Death associated protein kinases 1 and 2 (DAPK1/2), Caspase 6 (CASP6) and Endophilin-B1/BIF1 (SH3GLB1) were also identified as ATF4-interacting proteins.

### ATF4 target genes

Supplementary Table 2 shows 234 ATF4 gene targets that were identified in a systematic search of PubMed. Table 2 displays the 41 targets that were supported by two or more publications. Among the 45 articles that met the inclusion criteria explained in the methods section, 7 were high-throughput incorporating ChIP-seq with RNA-seq or transcriptome microarray (Han et al., 2013; Wang et al., 2015; Freundt et al., 2018; Chen et al., 2021; Örd et al., 2021; Ferguson et al., 2022; Zhong et al., 2022). Full utilisation of these results was not attained as only two articles provided a full results table (Han et al., 2013; Wang et al., 2015). The high-throughput articles listed provide Supplementary data, making it is possible to process the data for further information on ATF4 target genes.

ATF4 gene targets that were also identified as physical interactors of ATF4 were DDIT3/CHOP, TRIB3, CEBPB, CEBPD, CEBPG, ATF3, JDP2, and NFE2L1. Apart from TRIB3, these are all bZIP transcription factors. ATF5 and ATF6 are two more bZIP transcription factors found as ATF4 targets although they were not found to interact with ATF4. ATF4 targets that were characterised as implicated in apoptosis numbered 11, namely, BECN1, DPF2, G0S2, GHITM, MCL1, NLRP1, NOXA/PMAIP1, PUMA/BBC3, SNAI2, TP53BP2/ASPP2, and DNAJA3. Targets characterised as involved in autophagy are LC3B/MAP 1LC3B, ATG3, ATG7, SQSTM1/P62, BECN1, and WIPI1. Many target protein products that localise to mitochondria were identified; MTHFD2, GPT2, ALDH18A1, ALDH1L2, ALDH2, DNAJA3, GHITM, LONP1, PCK2, and TMEM11.

A nuclear exporter of tRNA, XPOT, and 15 tRNA synthetases were identified as ATF4 target genes; AARS, WARS, EPRS, GARS, IARS, LARS, NARS, SARS, VARS, YARS, CARS, FARSB, HARS, NARS, and TARS. Amino acid transporters; SLC7A11, SLC3A2, SLC7A1, SLC7A5, and SNAT2 were also identified as ATF4 target genes.

### Human ATF4 post-translational modifications

Figure 3 displays a graphical summary of human ATF4 PTMs that were amalgamated from three online databases that comprises both high-throughput proteomics and targeted low-throughput studies. Overall, the majority of ATF4 PTMs are concentrated in either the C-terminal bZIP domain or in a more N-terminal amino acid range 42–119. Phosphorylation of threonine residues T107, T114, T115, and T119 carried out by the protein kinase RET reduced transcription of apoptotic ATF4 target gene products NOXA and PUMA (Bagheri-Yarmand et al., 2015). These threonine phosphorylations are the only N-terminal modifications identified from a targeted low-throughput investigation. The other, predominantly ubiquitination, PTMs in the N-terminal cluster were all found in high-throughput proteomics studies. It was found K45 and K53 could be either SUMOylated or ubiquitinated while there was a phosphorylation detected at S69. Phosphorylation of S219 and S224 are required for βTRCP binding to cause ubiquitination of ATF4 to target it for proteasomal degradation (Pons et al., 2007). Ubiquitinations, SUMOylations, acetylations and phosphorylations were all identified in the C-terminal bZIP domain (Figure 3, References in Supplementary Table 3).

## Discussion

### ATF4-interactors and target gene regulation

While we identified a broad array of ATF4 target genes, it is clear that subsets of these genes will be only transcribed when ATF4 is bound to a specific binding partner. As ATF4 can heterodimerise with a range of other TFs, mostly bZIP domain containing (Table 1, Supplementary Table 1), it suggests ATF4 can bind to a variety of DNA sequences. By investigating DNA binding specificities of 270 bZIP transcription factor pairs, including ATF4 (Rodríguez-Martínez et al., 2017), it was discovered that 72% of bZIP heterodimer pairs bound sequences called conjoined half-sites – a DNA sequence which was an amalgam of each monomers’ binding site, i.e., an ATF4/ATF3 conjoined binding site would consist of half an ATF4 binding site followed by half an ATF3 binding site. This would suggest that the majority of ATF4 heterodimer binding sites could be predicted (assuming both the ATF4 and bZIP binding partner’s homodimeric binding sites were already well characterised). However, Rodríguez-Martínez et al. found some bZIP heterodimer’s DNA binding was found to be at ‘emergent’ or variably-spaced half-sites which could not have been predicted. It is also worth noting that Rodríguez-Martínez et al. were able to determine that many previous studies that were labouring under the impression that the ATF4 homodimers they were using had perhaps been working with impure ATF4·CEBPG heterodimers.

Our systematic review identified 234 putative ATF4 target genes (Supplementary Table 2) but this is likely to be a lower estimate on possible genes. An article using ChIP-seq in 3 T3-L1 mouse embryonic preadipocytes found ATF4 to bind 87,725 sites throughout the genome that were mapped to 16,164 genes (Chen et al., 2021). Coupled with RNA-seq, they reported 1,955 target genes that had both ATF4 occupancy and ATF4-dependent differential expression. Chen et al. also found ATF4 to interact with the chromatin architecture regulator CTCF, which may impart additional plasticity to the genome to facilitate this large-scale organisation of the transcriptome. Although a valuable resource, it is worth noting that Chen et al. identified far more binding sites than other high-throughput target gene investigations. The approach taken by Chen et al. may have an unspecific binding bias – this can occur with ChIP, particularly with a 1% formaldehyde cross-linking time of over 30 min (Baranello et al., 2016). However, Chen et al. specify in their methods that the 1% formaldehyde cross-linking took place for only 10 min which may reduce this. Two high-throughput ChIP-seq with RNA-seq or transcriptome microarray studies were found outside of the systematic PubMed search (Huggins et al., 2016; Tameire et al., 2019). Tameire et al. found ATF4 to co-occupy similar genomic regions as MYC at genes involved primarily in amino acid and protein synthesis. Huggins et al. found CEBPG-ATF4 heterodimers to be the predominant CARE-binding species in stressed mouse cells. Given ATF4’s large heterodimer DNA-binding capacity and disagreement in the literature, further high-throughput investigation into DNA binding sites is warranted. ATF4 could act to enable mRNA transcription from many genes during the ISR when translation of other TFs is limited. Alternatively, high amounts of ATF4 could saturate other TFs so they are unable to bind genes they would normally regulate.

Proteins that were found to both interact with ATF4 and their genes are targets of ATF4 are of interest. TRIB3, DDIT3, CEBPB, CEBPD, CEBPG, JDP2 and NFE2L1 meet this criteria. Of these, Tribbles homolog 3 (TRIB3) was the only protein identified that was not a bZIP TF. TRIB3 is a pseudokinase that is able to interact with ATF4, it inhibits transcriptional activity of ATF4, DDIT3 and CEBPB (Ord and Ord, 2017). DNA damage inducible transcript 3 (DDIT3) is a bZIP TF that is upregulated by the ISR and is involved in the induction of apoptosis alongside ATF4 (Pakos-Zebrucka et al., 2016; Wortel et al., 2017). DDIT3 and ATF4 heterodimers co-regulate many genes, the ATF4 target genes identified by Han et al. were acquired by Re-ChIP using ATF4 and DDIT3 antibodies (Han et al., 2013).

Activating transcription factor 3 (ATF3) is considered an integral part of the ISR (Jiang et al., 2004), it is a bZIP TF identified as a target and interactor of ATF4. Narita et al. showed that among 2 multiple myeloma cell lines and 6 primary samples, bortezomib treatment resulted in inconsistent protein induction of ATF4, DDIT3 and pro-apoptotic PMAIP1/NOXA but consistent ATF3 protein induction (Narita et al., 2015). ChIP-PCR showed consistent binding of ATF3 to *ATF3*, *ATF4*, *DDIT3*, and *PMAIP1* gene promoters. ATF4 binding to these promoters was inconsistent, however it is interesting that ATF4 was found to bind it’s own gene promoter in some cases. Furthermore, ATF3-ATF4 heterodimers were found to bind and upregulate mRNA expression from the gene for pro-apoptotic protein NOXA/PMAIP1 (Wang et al., 2009).

The CCAAT-enhancer-binding proteins (CEBPs) CEBPB, CEBPD, and CEBPG are bZIP TFs identified as dimerisation partners and target genes of ATF4. CEBPB is involved in immune responses, metabolism and it can induce cell-cycle arrest (reviewed in Niehrs and Calkhoven, 2020). CEBPB has involvement alongside ATF4 in regulating osteoblast differentiation (Tominaga et al., 2008). CEBPD has been found to promote transcription from the Prostaglandin-endoperoxide synthase 2 (PTGS2) promoter (Wang et al., 2006) and ATF4 has also been found to upregulate PTGS2 (Xiao et al., 2011; Di et al., 2018). CEBPG has been identified as a regulator of Interleukin-4 (Wang et al., 2006). Work in mice has reported CEBPG as the main ATF4 heterodimerisation partner to induce stress responsive genes (Huggins et al., 2016).

JUN dimerisation protein 2 (JDP2) is another bZIP TF identified as an ATF4 target gene and ATF4 dimerisation partner, it has been found to inhibit ATF4 transcriptional activity for some target genes including asparagine synthetase (ASNS; Engler et al., 2020). Nuclear factor erythroid-2-like 1 (NFE2L1) is a cap ‘n’ collar bZIP TF found to be a target gene and dimerisation partner of ATF4. It is endoplasmic reticulum-bound, where it functions as a cholesterol sensor and has been described as a guardian of cholesterol homeostasis (Widenmaier et al., 2017).

An outstanding question is to what extent different ATF4 dimerisation partners control particular subsets of ATF4 target genes. ATF4 dimerisation partners each will have their own regulation mechanisms. For example, ATF4 heterodimerisation partner JUN is a bZIP TF that is induced by a broad range of extracellular stimuli. JUN has been found to be required for cell-cycle progression and to protect cells against apoptosis (Wisdom et al., 1999). Thus, it may be that ATF4 heterodimer formation is governed by the relative amounts of these other transcription factors which are not directly influenced by the ISR. Alternatively, higher amounts of ATF4 could saturate other TFs, with ATF4 potentially changing or inhibiting their action. The majority of studies identifying ATF4 target genes induced ATF4 through endoplasmic reticulum stress with tunicamycin or thapsigargin. It was noted that some studies could identify genes as ATF4 targets with some ATF4 inducers but not others (Su and Kilberg, 2008; Chiang et al., 2013).

The endoplasmic reticulum (ER) stress induced unfolded protein response (UPR) activates the ISR and ATF4 through the eIF2α kinase PERK. However, ER stress induced UPR also activates other transcription factors such as XBP1 and ATF6 (Wu and Kaufman, 2006). XBP1 and ATF6 were both identified as ATF4 target genes in high-throughput studies (Supplementary Table 2). This combination of transcription factors could augment the ISR in comparison to, for example, amino acid starvation induced GCN2 activation. GCN2 activation has also been linked to induction of apoptosis (Gentz et al., 2013; Wang et al., 2018), showing apoptosis induction by the ISR is not specific to the UPR.

### ATF4 PTM regulation

Casein kinase 2 (CK2) has been highlighted as an important regulator of ATF4 and ATF4-interacting CEBP TFs (Wortel et al., 2017). CK2 was found to interact and phosphorylate ATF4 at Serine 215 (Ampofo et al., 2013; Siang et al., 2022). A S215A mutant caused a significant decrease in luciferase reporter activity under the control of two amino acid response elements or the promoter for ATF3 (Ampofo et al., 2013), suggesting S215 phosphorylation increases ATF4 activity. It was further shown that only the bZIP alpha helix of ATF4 is highly ordered and mutational studies showed the bZIP domain was required for CK2 to phosphorylate ATF4 in a disordered region (Siang et al., 2022). Knockdown of CK2-interacting ribosomal protein RPL41 resulted in a decrease in ATF4 ubiquitination and degradation, RPL41 was found to increase phosphorylation at βTRCP-recognising motif serine 219 (Wang A. et al., 2011).

ATF4 is targeted for ubiquitin-mediated proteasomal degradation by βTRCP, dependent on phosphorylation of ATF4 at serine 219 (Lassot et al., 2001). Recently, it has been shown in mouse pancreatic β-cells that phosphorylation of this serine residue on ATF4 is dependent on glycogen synthase kinase 3 (GSK3; Nagao et al., 2022). This is especially interesting given a lack of explanation for ATF4 upregulation from insulin signalling which inhibits GSK3 (Lewerenz et al., 2014).

RET kinase was found to phosphorylate ATF4 at threonine residues 107, 114, 115, and 119 and caused a decrease in transcription of pro-apoptotic ATF4 target genes NOXA and PUMA (Bagheri-Yarmand et al., 2015). However, it is unclear if these threonine phosphorylations cause a generic reduction of ATF4 transcriptional activity or if it could be more specific to these pro-apoptotic target genes. Interestingly, PNLIP, one of the few identified putative ATF4 target genes with downregulation associated with ATF4 gene binding was bound by a phosphorylated ATF4 (Park et al., 2019). Unfortunately, the authors do not state which phosphorylated ATF4 the antibody used was specific to. RSK2 induced phosphorylation of serine 245 on ATF4 was found to increase expression of osteocalcin (BGLAP; Yang et al., 2004).

ATF4 was found to be acetylated within the range of amino acids 270 to 300 by CREBBP (Gachon et al., 2002), CREBBP was also identified as an ATF4-interacting protein (Yukawa et al., 1999). The closely related protein EP300 was found to acetylate ATF4 at serine 311 (Lassot et al., 2005). EP300 associates with ATF4’s N-terminal to prevent ubiquitination but this effect was independent of EP300’s acetylation activity (Lassot et al., 2005). Figure 3 shows N-terminal ATF4 ubiquitinations, it is possible EP300 is blocking N-terminal ubiquitination through binding ATF4. An ubiquitin ligase substrate adaptor ASB7 (Uematsu et al., 2016) and an E3 ubiquitin ligase MDM2 (Girnita et al., 2003) were identified as ATF4 interactors (Supplementary Table 1), they could either be involved in ubiquitinating ATF4 at the N-terminal region.

### ATF4 and apoptosis

It has long been known that ATF4 is a key regulator of apoptosis induction (Han et al., 2013; Pakos-Zebrucka et al., 2016; Rajesh et al., 2016; Wortel et al., 2017). Supplementary Table 2 includes 11 ATF4 targets that were characterised as involved in regulation of apoptosis; what is clear from that list is that is no clear single route through which ATF4 appears to promote apoptosis – rather it pulls multiple potential levers to drive cells towards apoptosis.

Several ATF4 target genes were found to localise to the mitochondria which may act as an apoptosis signalling hub. The target gene DNAJA3/TID1 is a mitochondrial localised chaperone that has both short and long splicing variants. The long isoform of DNAJA3 for example can promote apoptosis whereas the short isoform supresses apoptosis (Syken et al., 1999). Furthermore, GHITM is a transmembrane mitochondrial protein that stimulates release of cytochrome c from mitochondria – a key stimulator of caspase 9 mediated apoptosis (Oka et al., 2008). In addition to this, gene regulation of Beclin-1 (BECN1) may be a route to apoptosis, as it can be cleaved by caspases leading to a C-terminal fragment localising with mitochondria and promoting apoptosis (Wirawan et al., 2010). We also identified ATF4 as interacting with Caspase 6 (CASP6), in a single high-throughput screen focused on neurodegeneration (Haenig et al., 2020). Caspase 6 is downstream of Caspases 3, 7 and 9 and is a protease that can promote the programmed cell death pathways apoptosis, necroptosis and pyroptosis (Zheng et al., 2020).

Cytochrome c release can also be driven through the BCL/BAX pathway, which is dependent on the disruption of BCL-2/BAX heterodimers and the resultant formation of BAX oligomers on the mitochondrial surface. One target gene of ATF4 is G0S2, a mitochondrial protein that prevents formation of BCL2-BAX heterodimers thereby promoting apoptosis through BAX oligomerisation (Welch et al., 2009). Additionally PUMA, was identified as an ATF4 target gene. PUMA promotes apoptosis by disrupting BAX/BAK heterodimers and promoting BAX oligomerisation (Yu and Zhang, 2008). NOXA was also found to be an ATF4 target gene and is a BH3-only Bcl-2 family member that targets the Bcl-2 family member, MCL1, for proteasomal degradation (Czabotar et al., 2007; MCL1 is a Bcl-2 family member that can exist in different isoforms by alterative splicing regulation; a long isoform promotes cell survival whereas a shorter isoform promotes apoptosis (Bingle et al., 2000)). ATF4 also targets TP53BP2 expression, a gene which interacts with both TP53 and BCL2 to facilitate apoptosis (Naumovski and Cleary, 1996). Interestingly, our review also identified Endophilin-B1/BIF1 as an ATF4 interacting protein. Endophilin-B1 interacts with apoptosis regulator BAX to play a pro-apoptotic role (Takahashi et al., 2005) – how an interaction with ATF4 may impact on the ability to carry out this role is unclear.

ATF4 was also found to interact directly with Death associated protein kinases 1 and 2 (DAPK2/3). DAPK2 and DAPK3 are two closely related serine/threonine protein kinases, of which DAPK3, also known as ZIP kinase, contains a leucine zipper and interacts with ATF4 (Kawai et al., 1998). The *DAPK2* gene can produce an evolutionary conserved isoform with a C-terminal leucine zipper, DAPK2β, that interacts with ATF4 (Shoval et al., 2011). Overexpression of DAPK3 has been found to induce apoptosis in NIH 3 T3 mouse embryonic fibroblast cells that was dependent on DAPK3’s kinase activity (Kawai et al., 1998). Kawai et al. highlight that ATF4 dimerisation with DAPK3 prevents DAPK3 homodimerisation and therefore inhibits its kinase activity-dependent apoptosis induction. Kawai et al. suggests that ATF4 is inhibiting apoptosis, however the ISR including ATF4 is capable of inducing apoptosis (Pakos-Zebrucka et al., 2016). An intriguing hypothetical possibility is that DAPK3 monomers could be normally rapidly targeted for degradation but DAPK3 monomer degradation is prevented through dimerisation with ATF4. This would allow a pool of ATF4-bound DAPK3 to build up within a cell. If there was subsequently a release of ATF4-bound DAPK3 monomers over a short period of time, it would allow high amounts of DAPK3 homodimers to form to promote apoptosis through DAPK3’s kinase activity.

Of interest Han et al., 2013 has proposed a model whereby a re-initiation of translation rather than ATF4/DDIT3 apoptosis-linked gene targets is the cause of apoptosis (Han et al., 2013). However, we characterised that three of their identified ATF4 and DDIT3 target genes (GHITM, DPF2 and DNAJA3) can be involved in apoptosis. Further work may be required to clarify whether the Han et al. model is correct.

### ATF4 and the cell-cycle

Several identified ATF4-interacting proteins (Supplementary Table 1) play a role in cellular division; CENPF, CEP83, SAPCD2, HAUS7, NDC80, LUZP1, and HOP2. Additional ATF4-interactors identified that are involved in cellular division but were identified outside of the systematic search are CEP290 (Sayer et al., 2006) and NEK6 (Vaz Meirelles et al., 2010). Research has linked ATF4 overexpression to cell-cycle arrest and apoptosis (Wu et al., 2017; Zong et al., 2017). However, research has found ATF4 to increase cancer cell line proliferation (Du et al., 2021; Wang et al., 2021). Additionally, knockdown of ATF4 in human cancer cell-mouse xenografts caused a large reduction in tumour mass compared to control cell xenografts (Ye et al., 2010). Nevertheless, it would be logical for ATF4 to inhibit cellular division in stressed proliferating cells. It is unclear how or in which way ATF4 could regulate cellular division but an enrichment of cell-cycle-linked ATF4-interacting proteins suggests ATF4 could directly regulate cell division. If ATF4 inhibits cellular division/cell-cycle progression then it is possible that cancer cells expressing high levels of ATF4 have found a way to circumvent this inhibition.

### ATF4 in the brain

Different cell types may have differential susceptibility to ATF4-induced apoptosis. This may apply to mature fully-differentiated neurons as they are generally non-dividing essential cells that cannot be replaced (Shadfar et al., 2022). There has been considerable interest in the role of ATF4 in the brain (Pitale et al., 2017; Costa-Mattioli and Walter, 2020). Supplementary Table 1 shows ATF4-interacting proteins that we identified as integral to neural synapses; GABBR1, GABBR2, DISC1, SNAP29, NLGN3, and APH1A. Further to this, ATF4 has been found localised to synapses (Lai et al., 2008) and it is established that the ISR including ATF4 inhibits the formation of long-term memory (Costa-Mattioli and Walter, 2020). As synaptic plasticity is theorised to be responsible for memory formation (Langille and Brown, 2018), it is possible that ATF4 may be involved in inhibition of synaptic plasticity. To support this, it has been noted that previous studies have found ATF4 to be a negative regulator of synaptic plasticity although ATF4 likely also plays a role in normal brain function (Pasini et al., 2015). It is also possible that through interacting with GABBR1/2 receptors, ATF4 could be activating the GABAergic system (Wu and Sun, 2015). As the GABAergic system inhibits neuronal activity (Vargas, 2018), ATF4 could be causing a general inhibition within the brain. Consistent with this, it was found that long-term knockdown of ATF4 in cultured rat hippocampal neurons significantly increased spontaneous action potentials and reduced GABBR1/2 activity (Corona et al., 2018). Overall, this could suggest that ATF4 can induce a quiescent protective state in neurons to protect them from damage during times of cellular stress. Schizophrenia is a condition in which synaptic plasticity is implicated (Mould et al., 2021). It is interesting to note that antipsychotic tranquiliser medications that are used to treat schizophrenia, such as olanzapine, are evidenced to induce cellular endoplasmic reticulum stress (He et al., 2019, 2021; Li W. et al., 2021; Zhou et al., 2022) that will induce ATF4 *via* the PERK-eIF2α pathway.

### Concluding remarks

The extensive options for ATF4 dimerisation partners illustrate the complexity of ATF4 regulation. PTMs provide an additional layer of complexity, perhaps further shaping ATF4 preferences for subsets of target genes. Furthermore, genome organisation *via* chromatin architecture and histone modifications surrounding target genes may alter ATF4 regulation and target specificity and may be the result of multiple diverse pathways which are distinct from the ISR and ATF4. The four eIF2α kinases that induce ATF4 through the ISR may have a role in tailoring ATF4 action through non-canonical substrate phosphorylation, but this has yet to be sufficiently explored. Throughout this review and literature search, it is apparent that the exact method of ISR induction, whether through UPR activation, starvation or tRNA synthetase inhibition, is likely to have very distinct downstream effects which may manifest in unique transcriptional responses by ATF4. This work summarises the various options available for ATF4 regulation, and the high degree of plasticity – what is still lacking is determining how ATF4 is guided to certain gene targets by upstream signalling.

What is also apparent is that the diversity of cell types used in various studies has produced an equally diverse set of results. The 234 gene targets we have identified may represent a ‘core’ portfolio of ATF4 targets which may be greatly augmented under certain conditions and in certain cell types. In one scenario, cellular apoptosis would be considered advantageous for a multicellular organism where irreparably stressed (for example) hepatic cells could be replaced. In contrast, extensive apoptosis in terminally-differentiated mature neurons that cannot be replaced would be detrimental to an organism. It is a reasonable conclusion therefore that the decision to commit to apoptosis will be cell-type-specific in higher eukaryotes. Comparative analysis of the regulation of ATF4 in apoptosis-able and apoptosis-unable cells may be a promising avenue to better identify the mechanisms controlling ATF4-mediated apoptosis. This knowledge may help in cancer research to identify cancerous cells that could have circumvented ATF4-mediated apoptosis or cell-cycle arrest.

## Author contributions

GN conducted the initial literature search and then drafted the manuscript. GM and GN then read manuscripts and refined inclusion criteria. GM contributed and edited the manuscript and created figures. All authors contributed to the article and approved the submitted version.

## Funding

This work was supported by the University of Dundee.

## Conflict of interest

The authors declare that the research was conducted in the absence of any commercial or financial relationships that could be construed as a potential conflict of interest.

## Publisher’s note

All claims expressed in this article are solely those of the authors and do not necessarily represent those of their affiliated organizations, or those of the publisher, the editors and the reviewers. Any product that may be evaluated in this article, or claim that may be made by its manufacturer, is not guaranteed or endorsed by the publisher.

## Supplementary material

The Supplementary material for this article can be found online at: https://www.frontiersin.org/articles/10.3389/fnmol.2023.1112253/full#supplementary-material
