# Supplementary material for: A stay of execution: ATF4 regulation and potential outcomes for the integrated stress response
Source: Front Mol Neurosci. 2023 Feb 7;16:1112253. doi: 10.3389/fnmol.2023.1112253 (PMC9941348; doi:10.3389/fnmol.2023.1112253)
Supplement: Supplementary file 1 [file Table_1.DOCX]

| ATF4 interacting protein | Brief description | References by species encoding ATF4 | Known to contain leucine zipper motif |
| --- | --- | --- | --- |
| **CEBPB** | bZIP transcription factor | **Human:** (Kawai et al., 1998) ; (Podust et al., 2001) ; (Tominaga et al., 2008) ; (Cohen et al., 2015). **Mouse:** (Vallejo et al., 1993) ; (Mann et al., 2013) ; (Ebert et al., 2020). **Unspecified:** (Vinson et al., 1993) ; (Lopez et al., 2007). | Yes |
| **JUN** | bZIP transcription factor | **Porcine:** (Kato et al., 1999). **Mouse:** (Chevray and Nathans, 1992) ; (Fung et al., 2007). **Unspecified:** (Hai and Curran, 1991) ; (Benbrook and Jones, 1994) ; (Steinmüller et al., 2001). | Yes |
| **CEBPG** | bZIP transcription factor | **Human:** (Su and Kilberg, 2008). **Mouse:** (Huggins et al., 2016) ; (Ebert et al., 2020). **Unspecified:** (Vinson et al., 1993) ; (Avitahl and Calame, 1994). | Yes |
| **ATF4** | bZIP transcription factor | **Human:** (Su and Kilberg, 2008). **Mouse:** (Vallejo et al., 1993) ; (Mann et al., 2013) ; (Ebert et al., 2020). | Yes |
| **CEBPE** | bZIP transcription factor | **Human:** (Chih et al., 2004) ; (Gombart et al., 2007). **Mouse:** (Chumakov et al., 2007). **Unspecified:** (Vinson et al., 1993). | Yes |
| **DDIT3** | bZIP transcription factor | **Human:** (Kawai et al., 1998) ; (Su and Kilberg, 2008). **Rat:** (Bromati et al., 2011). **Mouse:** (Gachon et al., 2001). | Yes |
| **CEBPA** | bZIP transcription factor | **Human:** (Kawai et al., 1998). **Mouse:** (Ebert et al., 2020). **Unspecified:** (Vinson et al., 1993). | Yes |
| **TXLNG** | LPS-stimulated | **Human:** (Su and Kilberg, 2008). **Mouse:** (Yu et al., 2008b).  **Unspecified:** (Yu et al., 2006). | Yes |
| **GABBR1** | GABA receptor | **Human:** (White et al., 2000). **Rat:** (Nehring et al., 2000) ; (Vernon et al., 2001). | No |
| **GABBR2** | GABA receptor | **Human:** (White et al., 2000). **Rat:** (Nehring et al., 2000) ; (Vernon et al., 2001). | No |
| **ATF3** | bZIP transcription factor | **Human:** (Kawai et al., 1998) ; (Su and Kilberg, 2008). | Yes |
| **CEBPD** | bZIP transcription factor | **Human:** (Kawai et al., 1998). **Mouse:** (Ebert et al., 2020). | Yes |
| **FOS** | bZIP transcription factor | **Mouse:** (Chevray and Nathans, 1992). **Unspecified:** (Hai and Curran, 1991). | Yes |
| **NFE2L2** | bZIP transcription factor | **Human:** (Su and Kilberg, 2008). **Rat**: (He et al., 2001). | Yes |
| **TAX** | Viral protein | **Human:** (Reddy et al., 1997) ; (Gachon et al., 1998). | No |
| **TRIB3** | Pseudokinase | **Human:** (Bowers et al., 2003). **Mouse:** (Örd and Örd, 2003). | No |
| **CAVIN1** | RNA Polymerase I transcript release factor | **Mouse:** (Ebert et al., 2020). | Yes |
| **CAVIN2** | Phospholipid-binding protein | **Mouse:** (Ebert et al., 2020). | Yes |
| **CREBZF** | bZIP transcription factor | **Unspecified:** (Hogan et al., 2006). | Yes |
| **DAPK3** | Death-associated protein kinase | **Human:** (Kawai et al., 1998). | Yes |
| **FRA1** | bZIP transcription factor | **Unspecified:** (Hai and Curran, 1991). | Yes |
| **HBZ** | Viral protein | **Unspecified:** (Gaudray et al., 2002). | Yes |
| **HOP2** | DNA binding protein | **Mouse:** (Zhang et al., 2019). | Yes |
| **JUNB** | bZIP transcription factor | **Human:** (Kawai et al., 1998). | Yes |
| **JUND** | bZIP transcription factor | **Human:** (Kawai et al., 1998). | Yes |
| **LUZP1** | Actin cytoskeleton-localising | **Mouse:** (Ebert et al., 2020). | Yes |
| **MAF** | bZIP transcription factor | **Mouse:** (Ebert et al., 2020). | Yes |
| **NFE2L1** | bZIP transcription factor | **Mouse:** (Murphy and Kolstø, 2000). | Yes |
| **TCF4** | Helix-loop-helix transcription factor | **Mouse:** (Muir et al., 2008). | Yes |
| **ABRO1** | BRISC complex subunit | **Mouse:** (Ambivero et al., 2012). | No |
| **BRD4-L** | Bromodomain and extraterminal domain protein | **Mouse:** (Ebert et al., 2020). | No |
| **CAVIN4** | Contains two coiled-coils | **Mouse:** (Ebert et al., 2020). | No |
| **CENPF** | Cell division regulation | **Human:** (Zhou et al., 2005). | No |
| **CREBBP** | Acetyltransferase transcriptional co-activator | **Mouse:** (Yukawa et al., 1999). | No |
| **DISC1** | Coiled coil rich C-terminal and N-terminal globular domain | **Human:** (Morris et al., 2003). | No |
| **ID2** | Helix-loop-helix transcription factor | **Mouse:** (Muir et al., 2008). | No |
| **LANA** | Viral protein | **Human:** (Lim et al., 2000). | No |
| **MYOD1** | Helix-loop-helix transcription factor | **Mouse:** (Muir et al., 2008). | No |
| **RPB11Bα** | RNA polymerase II subunit isoform | **Human:** (Proshkin et al., 2020). | No |
| **RPB3** | RNA polymerase II subunit | **Human:** (De Angelis et al., 2003). | No |
| **SCLERAXIS** | Helix-loop-helix transcription factor | **Mouse:** (Muir et al., 2008). | No |
| **SYNCOILIN** | Type III intermediate filament protein | **Mouse:** (Ebert et al., 2020). | No |
| **TCF15** | Helix-loop-helix transcription factor | **Mouse:** (Muir et al., 2008). | No |
| **TFIIAγ** | General transcription factor IIA subunit | **Mouse:** (Yu et al., 2008a). | No |

Supplementary Table 1: All reported ATF4-interacting proteins.
