# Supplementary material for: A stay of execution: ATF4 regulation and potential outcomes for the integrated stress response
Source: Front Mol Neurosci. 2023 Feb 7;16:1112253. doi: 10.3389/fnmol.2023.1112253 (PMC9941348; doi:10.3389/fnmol.2023.1112253)
Supplement: Supplementary file 2 [file Table_2.DOCX]

| Product of putative ATF4 target gene | Brief description (protein unless stated regulatory RNA) | Upregulation or Downregulation | References by species | Confidence  1=High  2=Medium  3=Low |
| --- | --- | --- | --- | --- |
| **DDIT3/CHOP** | bZIP transcription factor | Upregulation | **Human:** (Bruhat et al., 2007) ; (Shimizu et al., 2013) ; (Wang et al., 2015) ; (Lin et al., 2018) ; (Bagheri-Yarmand et al., 2019) ; (Örd et al., 2021). **Mouse:** (Chérasse et al., 2007) ; (Han et al., 2013) ; (Farooq et al., 2022). | 1 |
| **ASNS** | Asparagine Synthetase | Upregulation | **Human:** (Chen et al., 2004) ; (Su and Kilberg, 2008) ; (Gjymishka et al., 2009) ; (Burton et al., 2020) ; (Örd et al., 2021). **Mouse:** (Freundt et al., 2018). | 1 |
| **TRIB3** | Pseudokinase | Upregulation | **Human:** (Su and Kilberg, 2008) ; (Carraro et al., 2010) ; (Wang et al., 2015). **Rat:** (Bromati et al., 2011). **Mouse:** (Carraro et al., 2010); (Han et al., 2013). | 1 |
| **ATF3** | bZIP transcription factor | Upregulation | **Human:** (Pan et al., 2007) ; (Lee et al., 2013). **Rat:** (Zhou and Pan, 2011). **Mouse:** (Han et al., 2013) ; (Sasaki et al., 2020). | 1 |
| **VEGFA** | Vascular Endothelial Growth Factor | Upregulation | **Human:** (Su and Kilberg, 2008) ; (Wang et al., 2012) ; (Kim et al., 2020). **Mouse:** (Oskolkova et al., 2008) ; (Freundt et al., 2018). | 1 |
| **MTHFD2** | Mitochondrial bifunctional dehydrogenase | Upregulation | **Human:** (Wang et al., 2015) ; (Örd et al., 2021). **Mouse:** (Han et al., 2013) ; (Freundt et al., 2018). | 2 |
| **SLC7A11** | Cystine/glutamate antiporter xCT | Upregulation | **Human:** (Wang et al., 2015) ; (Örd et al., 2021) ; (Ferguson et al., 2022). **Mouse:** (Han et al., 2013). | 1 |
| **FGF21** | Fibroblast growth factor | Upregulation | **Human:** (Tao et al., 2022). **Mouse:** (Örd et al., 2018) ; (Sasaki et al., 2020). | 1 |
| **AARS** | Aminoacyl-tRNA synthetase | Upregulation | **Mouse:** (Han et al., 2013) ; (Shan et al., 2016) ; (Freundt et al., 2018). | 1 |
| **CEBPB** | bZIP transcription factor | Upregulation | **Human:** (Chen et al., 2005) ; (Wang et al., 2015). **Mouse:** (Guo et al., 2019). | 1 |
| **CHAC1** | Glutathione-specific gamma-glutamylcyclotransferase | Upregulation | **Human:** (Crawford et al., 2015) ; (Wang et al., 2015). **Mouse:** (Juliana et al., 2018). | 1 |
| **DDIT4/REDD1** | Negative regulator of mTOR | Upregulation | **Human:** (Wang et al., 2015) ; (Örd et al., 2021) ; (Han et al., 2021). | 2 |
| **GPT2** | Mitochondrial glutamic--pyruvic transaminase | Upregulation | **Human:** (Wang et al., 2015). **Mouse:** (Han et al., 2013) ; (Juliana et al., 2018). | 1 |
| **LC3B/ MAP1LC3B** | Autophagy protein | Upregulation | **Human:** (Shen et al., 2015) ; (Zhong et al., 2022). **Rat:** (Cai et al., 2022). | 1 |
| **PPP1R15A /GADD34** | Protein phosphatase 1 adaptor for eIF2 | Upregulation | **Human:** (Wang et al., 2015). **Mouse:** (Han et al., 2013) ; (Sasaki et al., 2020). | 1 |
| **PSAT1** | Phosphoserine aminotransferase | Upregulation | **Human:** (Gao et al., 2017) ; (Örd et al., 2021). **Mouse:** (Freundt et al., 2018). | 1 |
| **WARS** | Aminoacyl-tRNA synthetase | Upregulation | **Human:** (Wang et al., 2015). **Mouse:** (Han et al., 2013) ; (Shan et al., 2016). | 1 |
| **ALDH18A1** | Mitochondrial aldehyde dehydrogenase | Upregulation | **Mouse:** (Han et al., 2013) ; (Freundt et al., 2018). | 2 |
| **ATG7** | Autophagy related protein | Upregulation | **Human:** (Zhong et al., 2022). **Rat:** (Cai et al., 2022). | 2 |
| **BGLAP (Osteocalcin)** | Bone gamma-carboxyglutamic acid-containing protein | Upregulation | **Mouse:** (Tominaga et al., 2008) ; (Yu et al., 2009). | 2 |
| **CDSN** | Corneodesmosin | Upregulation | **Mouse:** (Han et al., 2013) ; (Sasaki et al., 2020). | 2 |
| **EIF2S2** | eIF2 subunit (eIF2β) | Upregulation | **Mouse:** (Han et al., 2013) ; (Freundt et al., 2018). | 2 |
| **EPRS** | Aminoacyl-tRNA synthetase | Upregulation | **Mouse:** (Han et al., 2013) ; (Shan et al., 2016). | 2 |
| **FGF19** | Fibroblast growth factor | Upregulation | **Human:** (Shimizu et al., 2013) ; (Lang et al., 2021). | 2 |
| **GARS** | Aminoacyl-tRNA synthetase | Upregulation | **Mouse:** (Han et al., 2013) ; (Shan et al., 2016). | 2 |
| **GDF15** | Growth/differentiation factor | Upregulation | **Human:** (Wang et al., 2015) ; (Li et al., 2021). | 2 |
| **HERPUD1** | Homocysteine-responsive ER-resident ubiquitin-like domain | Upregulation | **Human:** (Wang et al., 2015). **Mouse:** (Freundt et al., 2018). | 2 |
| **HSPA5** | Molecular chaperone | Upregulation | **Human:** (Wang et al., 2015). **Mouse:** (Han et al., 2013). | 2 |
| **IARS** | Aminoacyl-tRNA synthetase | Upregulation | **Mouse:** (Han et al., 2013) ; (Shan et al., 2016). | 2 |
| **JDP2** | bZIP transcription factor | Upregulation | **Human:** (Wang et al., 2015). **Mouse:** (Freundt et al., 2018). | 2 |
| **KDM7A** | Histone lysine demethylase | Upregulation | **Human:** (Wang et al., 2015). **Mouse:** (Han et al., 2013). | 2 |
| **LARS** | Aminoacyl-tRNA synthetase | Upregulation | **Mouse:** (Han et al., 2013) ; (Shan et al., 2016). | 2 |
| **miR-552** | Micro RNA 552 | Upregulation | **Human:** (Feng et al., 2022). **Mouse:** (Feng et al., 2022). | 2 |
| **MKNK2** | MAP kinase-interacting serine/threonine-protein kinase | Upregulation | **Human:** (Wang et al., 2015) ; (Bagheri-Yarmand et al., 2019). | 2 |
| **NARS** | Aminoacyl-tRNA synthetase | Upregulation | **Mouse:** (Han et al., 2013) ; (Shan et al., 2016). | 2 |
| **PTGS2/COX2** | Prostaglandin-endoperoxide synthase | Upregulation | **Human:** (Xiao et al., 2011) ; (Di et al., 2018). | 2 |
| **SARS** | Aminoacyl-tRNA synthetase | Upregulation | **Mouse:** (Han et al., 2013) ; (Shan et al., 2016). | 2 |
| **SQSTM1/P62** | Autophagosome cargo protein | Upregulation | **Rat:** (Cai et al., 2022). **Mouse:** (Han et al., 2013). | 2 |
| **VARS** | Aminoacyl-tRNA synthetase | Upregulation | **Mouse:** (Han et al., 2013) ; (Shan et al., 2016). | 2 |
| **VLDLR** | Very-low-density-lipoprotein receptor | Upregulation | **Human:** (Wang et al., 2015). **Mouse:** (Han et al., 2013). | 2 |
| **YARS** | Aminoacyl-tRNA synthetase | Upregulation | **Mouse:** (Han et al., 2013) ; (Shan et al., 2016). | 2 |
| **2610528E23RIK** | Secreted protein | Upregulation | **Mouse:** (Han et al., 2013). | 2 |
| **2700007P21RIK** | Histone deacetylase | Upregulation | **Mouse:** (Han et al., 2013). | 2 |
| **A630007B06RIK** | Coiled-coil domain-containing protein | Upregulation | **Mouse:** (Han et al., 2013). | 2 |
| **ABCC4** | ATP-dependent transporter | Upregulation | **Mouse:** (Han et al., 2013). | 2 |
| **ABRAXAS1** | BRCA1-A complex subunit | Upregulation | **Mouse:** (Han et al., 2013). | 2 |
| **ACOT2** | Hydrolyses Coenzyme A esters | Upregulation | **Mouse:** (Han et al., 2013). | 2 |
| **ADORA2B** | Adenosine receptor | Upregulation | **Human:** (Wang et al., 2015). | 2 |
| **AGAP1** | GTPase-activating protein | Upregulation | **Mouse:** (Han et al., 2013). | 2 |
| **AKNA** | Centrosomal protein | Upregulation | **Mouse:** (Han et al., 2013). | 2 |
| **ALDH1L2** | Mitochondrial aldehyde dehydrogenase | Upregulation | **Mouse:** (Han et al., 2013). | 2 |
| **ALDH2** | Mitochondrial aldehyde dehydrogenase | Upregulation | **Human:** (Wang et al., 2015). | 2 |
| **ALOXE3** | Epidermis-type lipoxygenase 3 | Upregulation | **Human:** (Wang et al., 2015). | 2 |
| **ANGPTL3** | Angiopoietin-related protein | Upregulation | **Mouse:** (Zhao et al., 2015). | 2 |
| **ANGPTL4** | Angiopoietin-related protein | Upregulation | **Mouse:** (Khozoie et al., 2012). | 2 |
| **ANGPTL6** | Angiopoietin-related protein | Upregulation | **Mouse:** (Han et al., 2013). | 2 |
| **AP3S2** | AP-3 complex subunit | Upregulation | **Human:** (Wang et al., 2015). | 2 |
| **APOE** | Lipid binding | Contradictory | **Human:** (Geng et al., 2011). | 3 |
| **AR** | Androgen receptor | Downregulation | **Human:** (Li et al., 2022). | 2 |
| **ARHGDIA** | Rho GDP-dissociation inhibitor | Upregulation | **Rat:** (Pasini et al., 2016). | 2 |
| **ARHGEF2** | Rho guanine nucleotide exchange factor | Upregulation | **Mouse:** (Han et al., 2013). | 2 |
| **ARNTL** | Aryl hydrocarbon receptor nuclear translocator-like protein | Upregulation | **Human:** (Wang et al., 2015). | 2 |
| **ASB1** | Ankyrin repeat and SOCS box protein | Upregulation | **Human:** (Wang et al., 2015). | 2 |
| **ASCC2** | ASCC complex subunit | Upregulation | **Mouse:** (Han et al., 2013). | 2 |
| **ATAD2** | ATPase family AAA domain-containing protein | Upregulation | **Mouse:** (Freundt et al., 2018). | 2 |
| **ATF5** | bZIP transcription factor | Upregulation | **Mouse:** (Han et al., 2013). | 2 |
| **ATF6** | bZIP transcription factor | Upregulation | **Mouse:** (Han et al., 2013). | 2 |
| **ATG10** | Autophagy-related protein | Upregulation | **Rat:** (Cai et al., 2022). | 3 |
| **ATG3** | Autophagy-related protein | Upregulation | **Rat:** (Cai et al., 2022). | 2 |
| **ATG5** | Autophagy-related protein | Upregulation | **Rat:** (Cai et al., 2022). | 3 |
| **ATOH8** | Basic helix-loop-helix transcription factor | Upregulation | **Mouse:** (Kitakaze et al., 2021). | 2 |
| **BBS10** | Probable molecular chaperone | Upregulation | **Human:** (Wang et al., 2015). | 2 |
| **BCAT2** | Branched-chain amino acid transaminase | Upregulation | **Mouse:** (Han et al., 2013). | 2 |
| **BECN1** | Beclin-1. Involved in autophagy and apoptosis | Upregulation | **Rat:** (Cai et al., 2022). | 2 |
| **BRF2** | Transcription factor IIIB subunit | Upregulation | **Human:** (Wang et al., 2015). | 2 |
| **CA9** | Carbonic anhydrase | Upregulation | **Human:** (van den Beucken et al., 2009). | 2 |
| **CARS** | Aminoacyl-tRNA synthetase | Upregulation | **Mouse:** (Shan et al., 2016). | 2 |
| **CBX4** | E3 SUMO-protein ligase | Upregulation | **Human:** (Wang et al., 2015). | 2 |
| **CCDC9** | Coiled-coil domain-containing protein | Upregulation | **Human:** (Wang et al., 2015). | 2 |
| **CD74** | Antigen | Upregulation | **Mouse:** (Han et al., 2013). | 2 |
| **CDCP1** | CUB domain-containing protein | Upregulation | **Human:** (Wang et al., 2015). | 2 |
| **CDH24** | Cadherin | Upregulation | **Mouse:** (Han et al., 2013). | 2 |
| **CEBPD** | bZIP transcription factor | Upregulation | **Mouse:** (Chen et al., 2021). | 2 |
| **CEBPG** | bZIP transcription factor | Upregulation | **Mouse:** (Han et al., 2013). | 2 |
| **CLCN3** | H+/Cl- exchange transporter | Upregulation | **Mouse:** (Han et al., 2013). | 2 |
| **CLTCL1** | Clathrin heavy chain | Upregulation | **Human:** (Wang et al., 2015). | 2 |
| **CNPPD1** | Single-pass membrane protein | Upregulation | **Mouse:** (Han et al., 2013). | 2 |
| **CRISPLD2** | Cysteine-rich secretory protein LCCL domain-containing | Upregulation | **Human:** (Wang et al., 2015). | 2 |
| **CSTF2T** | Cleavage stimulation factor subunit 2 tau variant | Upregulation | **Human:** (Wang et al., 2015). | 2 |
| **CTCF** | Regulator of chromatin architecture | Upregulation | **Mouse:** (Chen et al., 2021). | 2 |
| **CXADR** | Cell surface receptor | Upregulation | **Mouse:** (Han et al., 2013). | 2 |
| **CYB5R1** | Metabolic enzyme | Upregulation | **Mouse:** (Han et al., 2013). | 2 |
| **DDR2** | Discoidin domain receptor tyrosine kinase | Upregulation | **Mouse:** (Han et al., 2013). | 2 |
| **DLGAP1-AS1** | DLGAP1 antisense non-coding RNA | Upregulation | **Human:** (Wang et al., 2015). | 2 |
| **DNAJA3** | Apoptosis-linked mitochondrial protein | Upregulation | **Mouse:** (Han et al., 2013). | 2 |
| **DNAJB1** | DnaJ homolog subfamily B member | Upregulation | **Human:** (Wang et al., 2015). | 2 |
| **DNAJB5** | DnaJ homolog subfamily B member | Upregulation | **Human:** (Wang et al., 2015). | 2 |
| **DPF2** | Apoptosis-linked zinc finger protein | Upregulation | **Mouse:** (Han et al., 2013). | 2 |
| **DPYSL2** | Dihydropyrimidinase-related protein | Upregulation | **Human:** (Wang et al., 2015). | 2 |
| **DUSP1** | Dual specificity phosphatase | Upregulation | **Human:** (Hocsak et al., 2017). | 2 |
| **DUSP6** | Dual specificity phosphatase | Downregulation | **Mouse:** (Han et al., 2013). | 2 |
| **DYSF** | Dystrophy-associated fer-1-like protein | Downregulation | **Mouse:** (Han et al., 2013). | 2 |
| **EIF3C** | eIF3 subunit | Upregulation | **Mouse:** (Han et al., 2013). | 2 |
| **EMILIN1** | Extracellular matrix glycoprotein | Downregulation | **Mouse:** (Han et al., 2013). | 2 |
| **EPO** | Glycoprotein cytokine | Contradictory | **Human:** (Chiang et al., 2013). | 3 |
| **ERLIN1** | ER lipid raft-associated protein | Upregulation | **Mouse:** (Han et al., 2013). | 2 |
| **ERO1L** | Oxidoreductase | Upregulation | **Mouse:** (Han et al., 2013). | 2 |
| **EVI2A** | Ecotropic viral integration site 2A protein homolog | Upregulation | **Human:** (Wang et al., 2015). | 2 |
| **FADS3** | Fatty acid desaturase | Upregulation | **Mouse:** (Han et al., 2013). | 2 |
| **FAM102A** | Induced by estrogen | Upregulation | **Human:** (Wang et al., 2015). | 2 |
| **FAM27C** | Long non-coding RNA | Upregulation | **Human:** (Wang et al., 2015). | 2 |
| **FARSB** | Aminoacyl-tRNA synthetase | Upregulation | **Mouse:** (Shan et al., 2016). | 2 |
| **FMNL1** | Formin-like protein | Upregulation | **Human:** (Wang et al., 2015). | 2 |
| **G0S2** | Promotes apoptosis by binding to BCL2 | Upregulation | **Mouse:** (Ma et al., 2019). | 2 |
| **GADD45A** | Growth arrest and DNA-damage-inducible | Upregulation | **Human:** (Wang et al., 2015). | 2 |
| **GEMIN7** | SMN complex component found in Gems of Cajal bodies | Upregulation | **Human:** (Wang et al., 2015). | 2 |
| **GFPT1** | Glutamine--fructose-6-phosphate aminotransferase | Upregulation | **Human:** (Wang et al., 2015). | 2 |
| **GHITM** | Mitochondrial Bcl-2-interacting protein | Upregulation | **Mouse:** (Han et al., 2013). | 2 |
| **GNPNAT1** | Glucosamine 6-phosphate acetylase | Upregulation | **Mouse:** (Han et al., 2013). | 2 |
| **GPAT3** | Glycerol-3-phosphate acyltransferase | Upregulation | **Human:** (Wang et al., 2015). | 2 |
| **GTPBP2** | GTP-binding protein | Upregulation | **Mouse:** (Han et al., 2013). | 2 |
| **HARS** | Aminoacyl-tRNA synthetase | Upregulation | **Mouse:** (Shan et al., 2016). | 2 |
| **HAS2** | Hyaluronan synthase | Downregulation | **Mouse:** (Han et al., 2013). | 2 |
| **HDAC1** | Histone deacetylase | Upregulation | **Human:** (Zeng et al., 2016). | 2 |
| **HDAC8** | Histone lysine deacetylase | Upregulation | **Mouse:** (Han et al., 2013). | 2 |
| **HFE** | Iron regulatory protein | Upregulation | **Mouse:** (Han et al., 2013). | 2 |
| **HYOU1** | Hypoxia upregulated protein | Upregulation | **Human:** (Zong et al., 2016). | 2 |
| **ICK** | Serine/threonine-protein kinase | Upregulation | **Mouse:** (Han et al., 2013). | 2 |
| **IFT172** | Intraflagellar transport protein | Upregulation | **Mouse:** (Han et al., 2013). | 2 |
| **IHH** | Cell signalling protein | Upregulation | **Mouse:** (Piao et al., 2013). | 2 |
| **IL2** | Cytokine Interleukin 2 | Upregulation | **Mouse:** (Chang et al., 2012). | 2 |
| **JAG1** | Ligand for Notch receptors | Upregulation | **Mouse:** (Liu et al., 2022). | 2 |
| **KCTD14** | Potassium channel tetramerisation domain containing protein | Upregulation | **Human:** (Wang et al., 2015). | 2 |
| **KDM6B** | Lysine demethylase | Upregulation | **Human:** (Shan et al., 2012). | 2 |
| **KLF9** | Krueppel-like transcription factor | Upregulation | **Human:** (Bagheri-Yarmand et al., 2019). | 2 |
| **KRTCAP2** | OST complex subunit | Upregulation | **Mouse:** (Han et al., 2013). | 2 |
| **LAMP3** | Lysosome-associated membrane glycoprotein | Upregulation | **Human:** (Burton et al., 2020). | 2 |
| **LARP1** | Translational inhibitor of terminal oligopyrimidine mRNAs | Upregulation | **Mouse:** (Farooq et al., 2022). | 2 |
| **LECT2** | Leukocyte cell-derived chemotaxin-2 | Upregulation | **Human:** (Park et al., 2021). | 2 |
| **LEPROTL1** | Leptin receptor overlapping transcript-like | Upregulation | **Mouse:** (Han et al., 2013). | 2 |
| **LGALS3** | Carbohydrate-binding protein | Upregulation | **Mouse:** (Han et al., 2013). | 2 |
| **LONP1** | Mitochondrial serine protease | Upregulation | **Mouse:** (Han et al., 2013). | 2 |
| **LURAP1L** | Leucine rich adaptor protein 1-like | Upregulation | **Mouse:** (Han et al., 2013). | 2 |
| **LZTFL1** | Leucine zipper transcription factor-like | Upregulation | **Human:** (Bagheri-Yarmand et al., 2019). | 2 |
| **MALAT1** | Conserved regulatory long non-coding RNA | Upregulation | **Human:** (Wang et al., 2015). | 2 |
| **MARS** | Aminoacyl-tRNA synthetase | Upregulation | **Mouse:** (Shan et al., 2016). | 2 |
| **MCL1** | Bcl-2 family protein | Upregulation | **Human:** (Hu et al., 2012). | 2 |
| **miR-145** | Micro RNA 145 | Upregulation | **Human:** (Zhao et al., 2022). | 2 |
| **MKX** | Homeobox transcription factor | Upregulation | **Human:** (Wang et al., 2015). | 2 |
| **MLPH** | Membrane transport protein Melanophilin | Upregulation | **Human:** (Wang et al., 2015). | 2 |
| **MOCS3** | Adenylyltransferase and sulfurtransferase | Upregulation | **Human:** (Wang et al., 2015). | 2 |
| **MTA1** | Metastasis-associated protein | Upregulation | **Human:** (Zeng et al., 2016). | 2 |
| **MTM1** | Lipid phosphatase | Upregulation | **Mouse:** (Han et al., 2013). | 2 |
| **NBR2** | AMPK-interacting long non-coding RNA | Upregulation | **Human:** (Wang et al., 2015). | 2 |
| **NCOA7** | Nuclear receptor coactivator | Upregulation | **Human:** (Wang et al., 2015). | 2 |
| **NFE2L1** | bZIP transcription factor | Upregulation | **Mouse:** (Han et al., 2013). | 2 |
| **NFIL3** | Nuclear factor interleukin-3-regulated | Upregulation | **Human:** (Wang et al., 2015). | 2 |
| **NFU1** | Iron-sulphur cluster scaffold protein | Upregulation | **Mouse:** (Han et al., 2013). | 2 |
| **NLRP1** | Inflammasome and apoptosis-linked | Upregulation | **Human:** (D’Osualdo et al., 2015). | 2 |
| **NNMT** | Nicotinamide N-methyltransferase | Upregulation | **Mouse:** (Song et al., 2020). | 2 |
| **NOXA/PMAIP1** | Pro-apoptotic Bcl-2 family protein | Upregulation | **Human:** (Bagheri-Yarmand et al., 2015). | 2 |
| **NRIP2** | Nuclear receptor-interacting protein | Upregulation | **Mouse:** (Han et al., 2013). | 2 |
| **NUPR1** | Apoptosis-linked Nuclear protein | Upregulation | **Mouse:** (Freundt et al., 2018). | 2 |
| **OTUB2** | Deubiquitinating cysteine protease | Upregulation | **Mouse:** (Han et al., 2013). | 2 |
| **P22PHOX/CYBA** | Neutrophil cytochrome b light chain | Upregulation | **Human:** (Petry et al., 2019). | 2 |
| **PACSIN2** | Lipid-binding protein | Upregulation | **Mouse:** (Han et al., 2013). | 2 |
| **PAQR3** | Transmembrane receptor | Upregulation | **Mouse:** (Han et al., 2013). | 2 |
| **PCK2** | Mitochondrial phosphoenolpyruvate carboxykinase | Upregulation | **Human:** (Wang et al., 2015). | 2 |
| **PHYHD1** | 2-oxoglutarate-dependent dioxygenase | Upregulation | **Mouse:** (Han et al., 2013). | 2 |
| **PLIN2** | Lipid-binding protein | Upregulation | **Mouse:** (Khozoie et al., 2012). | 2 |
| **PNLIP** | Pancreatic triacylglycerol lipase | Downregulation | **Mouse:** (Park et al., 2019). | 2 |
| **PNRC2** | Proline-rich nuclear receptor coactivator | Upregulation | **Mouse:** (Han et al., 2013). | 2 |
| **PPARG** | Peroxisome proliferator- activated receptor gamma | Upregulation | **Mouse:** (Chen et al., 2021). | 2 |
| **PRDM15** | PR domain zinc finger protein | Upregulation | **Mouse:** (Han et al., 2013). | 2 |
| **PRICKLE1** | Nuclear receptor | Upregulation | **Mouse:** (Khozoie et al., 2012). | 2 |
| **PSEN1** | Gamma secretase catalytic subunit | Upregulation | **Human:** (Mitsuda et al., 2007). | 2 |
| **PTN** | Neurite growth-promoting factor | Downregulation | **Mouse:** (Han et al., 2013). | 2 |
| **PUMA/BBC3** | Pro-apoptotic Bcl-2 family protein | Upregulation | **Human:** (Bagheri-Yarmand et al., 2019). | 2 |
| **RANKL** | Tumor necrosis factor family cytokine | Upregulation | **Mouse:** (Baek et al., 2017). | 2 |
| **RGMB** | Repulsive guidance molecule | Upregulation | **Human:** (Wang et al., 2015). | 2 |
| **RHBDD1** | Rhomboid-related protein | Upregulation | **Mouse:** (Freundt et al., 2018). | 2 |
| **RHBDD2** | Rhomboid domain-containing protein | Upregulation | **Human:** (Wang et al., 2015). | 2 |
| **RND3** | Rho-related GTP-binding protein | Upregulation | **Human:** (Wang et al., 2015). | 2 |
| **RUNX2** | Runt-related transcription factor | Upregulation | **Human:** (Wang et al., 2015). | 2 |
| **S100A6** | Calcium-binding protein | Downregulation | **Mouse:** (Freundt et al., 2018). | 2 |
| **SESN2** | Leucine sensor Sestrin2 | Upregulation | **Human:** (Wang et al., 2015). | 2 |
| **SETX** | Probable RNA/DNA helicase | Upregulation | **Human:** (Ramachandran et al., 2021). | 2 |
| **SIAH1** | E3 ubiquitin-protein ligase | Upregulation | **Human:** (Bagheri-Yarmand et al., 2019). | 2 |
| **SLC20A1** | Sodium-phosphate symporter | Upregulation | **Mouse:** (Han et al., 2013). | 2 |
| **SLC3A2** | Amino acid transporter LAT1 subunit | Upregulation | **Human:** (Wang et al., 2015). | 2 |
| **SLC7A1** | High affinity cationic amino acid transporter | Upregulation | **Mouse:** (Juliana et al., 2018). | 2 |
| **SLC7A5** | Amino acid transporter LAT1 subunit | Upregulation | **Mouse:** (Han et al., 2013). | 2 |
| **SNAI2** | Apoptosis-linked zinc finger protein | Upregulation | **Mouse:** (Han et al., 2013). | 2 |
| **SNAI3** | Zinc finger protein | Upregulation | **Mouse:** (Khozoie et al., 2012). | 2 |
| **SNAT2** | Sodium-coupled neutral amino acid transporter | Upregulation | **Human:** (Su and Kilberg, 2008). | 2 |
| **SNCG** | Synuclein Gamma | Upregulation | **Human:** (Hua et al., 2009). | 2 |
| **SNORD83B** | Small nucleolar RNA | Upregulation | **Human:** (Wang et al., 2015). | 2 |
| **SNORD96A** | Small nucleolar RNA | Upregulation | **Human:** (Wang et al., 2015). | 2 |
| **SREBP1** | Sterol regulatory element-binding protein | Upregulation | **Human:** (Xu et al., 2021). | 2 |
| **SREBP2** | Sterol regulatory element-binding protein | Upregulation | **Human:** (Xu et al., 2021). | 2 |
| **SS18L2** | Nuclear protein | Upregulation | **Mouse:** (Han et al., 2013). | 2 |
| **STC2** | Secreted homodimeric glycoprotein | Upregulation | **Mouse:** (Han et al., 2013). | 2 |
| **STEAP1** | Metalloreductase | Upregulation | **Mouse:** (Han et al., 2013). | 2 |
| **STOX1** | Storkhead-box protein | Upregulation | **Human:** (Wang et al., 2015). | 2 |
| **SYNCYTIN2** | Endogenous retroviral envelope protein | Upregulation | **Human:** (Toufaily et al., 2015). | 2 |
| **TAB2** | MAP3K7-interacting protein | Upregulation | **Mouse:** (Han et al., 2013). | 2 |
| **TAF15** | TATA-binding protein-associated factor | Upregulation | **Mouse:** (Han et al., 2013). | 2 |
| **TARS** | Aminoacyl-tRNA synthetase | Upregulation | **Mouse:** (Shan et al., 2016). | 2 |
| **TBPL1** | TATA box-binding protein-like protein | Upregulation | **Mouse:** (Han et al., 2013). | 2 |
| **TES** | Scaffold protein | Upregulation | **Human:** (Wang et al., 2015). | 2 |
| **TIPIN** | DNA replication-linked, TIMELESS-interacting | Upregulation | **Human:** (Wang et al., 2015). | 2 |
| **TLR2** | Toll-like receptor | Upregulation | **Mouse:** (Liao et al., 2016). | 2 |
| **TMEM11** | Mitochondrial transmembrane protein | Upregulation | **Mouse:** (Han et al., 2013). | 2 |
| **TNFAIP6** | Hyaluronan-binding protein | Upregulation | **Bovine:** (Sayasith et al., 2008). | 3 |
| **TP53BP2/ASPP2** | Apoptosis-stimulating of p53 protein | Upregulation | **Rat:** (Zhou et al., 2021). | 2 |
| **TSC22D3** | TSC22 domain family protein | Upregulation | **Human:** (Wang et al., 2015). | 2 |
| **TSPYL4** | Nucleosome assembly protein | Upregulation | **Mouse:** (Han et al., 2013). | 2 |
| **TUFT1** | Acidic phosphorylated glycoprotein Tuftelin | Upregulation | **Human:** (Wang et al., 2015). | 2 |
| **TXNL4B** | Thioredoxin-like protein | Upregulation | **Human:** (Wang et al., 2015). | 2 |
| **TXNRD1** | Thioredoxin reductase | Upregulation | **Human:** (Wang et al., 2015). | 2 |
| **UBE2G2** | Ubiquitin-conjugating enzyme | Upregulation | **Mouse:** (Han et al., 2013). | 2 |
| **WDR36** | WD repeat-containing protein | Upregulation | **Human:** (Wang et al., 2015). | 2 |
| **WIPI1** | Autophagy-involved. WD repeat domain phosphoinositide-interacting protein | Upregulation | **Human:** (Wang et al., 2015). | 2 |
| **XBP1** | UPR transcription factor X-box-binding protein | Upregulation | **Human:** (Wang et al., 2015). | 2 |
| **XPOT** | tRNA nuclear exporter | Upregulation | **Mouse:** (Han et al., 2013). | 2 |
| **YPEL5** | GID/CTLH E3 ubiquitin-ligase subunit | Upregulation | **Mouse:** (Han et al., 2013). | 2 |
| **ZBTB18** | Zinc finger and BTB domain-containing protein | Upregulation | **Mouse:** (Han et al., 2013). | 2 |
| **ZBTB38** | Zinc finger and BTB domain-containing protein | Upregulation | **Mouse:** (Cai et al., 2017). | 2 |
| **ZC3HAV1** | Antiviral zinc finger protein | Upregulation | **Mouse:** (Han et al., 2013). | 2 |
| **ZFP69B** | Zinc finger protein | Upregulation | **Human:** (Wang et al., 2015). | 2 |
| **ZNF268** | Zinc finger protein | Upregulation | **Human:** (Guo et al., 2006). | 2 |
| **ZNF419** | Zinc finger protein | Upregulation | **Human:** (Wang et al., 2015). | 2 |
| **ZNF598** | E3 ubiquitin-protein ligase | Upregulation | **Mouse:** (Han et al., 2013). | 2 |
| **ZNF682** | Zinc finger protein | Upregulation | **Human:** (Wang et al., 2015). | 2 |

**Supplementary Table 2: All reported ATF4 Gene targets.**
