# Supplementary material for: A stay of execution: ATF4 regulation and potential outcomes for the integrated stress response
Source: Front Mol Neurosci. 2023 Feb 7;16:1112253. doi: 10.3389/fnmol.2023.1112253 (PMC9941348; doi:10.3389/fnmol.2023.1112253)
Supplement: Supplementary file 3 [file Table_3.DOCX]

| **Residue** | **Modification** | **Source(s)** |
| --- | --- | --- |
| K42 | Ubiquitination | Thebiogrid (TBG) |
| K45 | Ubiquitination | TBG, PhosphoSite Plus (PSP) |
| K45 | Sumoylation | PSP |
| K53 | Ubiquitination | TBG, PSP |
| K53 | Glycyl lysine isopeptide | Uniprot |
| K55 | Ubiquitination | PSP, TBG |
| S69 | Phosphorylation | PSP |
| K75 | Ubiquitination | PSP, TBG |
| K88 | Ubiquitination | PSP, TBG |
| K92 | Ubiquitination | PSP, TBG |
| T107 | Phosphorylation | PSP |
| T114 | Phosphorylation | PSP |
| T115 | Phosphorylation | PSP |
| T119 | Phosphorylation | PSP |
| S184 | Phosphorylation | PSP |
| T213 | Phosphorylation | Uniprot |
| S215 | Phosphorylation | Uniprot, PSP |
| S219 | Phosphorylation | Uniprot, PSP |
| S224 | Phosphorylation | Uniprot, PSP |
| S231 | Phosphorylation | Uniprot |
| S235 | Phosphorylation | Uniprot |
| P236 | 4-hydroxyproline | Uniprot |
| S245 | Phosphorylation | Uniprot, PSP |
| S248 | Phosphorylation | Uniprot, PSP |
| L252 | Phosphorylation | PSP |
| K259 | Ubiquitination | PSP |
| K259 | Glycyl lysine isopeptide | Uniprot |
| K267 | Sumoylation | PSP |
| K267 | Glycyl lysine isopeptide | Uniprot |
| K267 | Ubiquitination | TBG |
| K272 | Ubiquitination | TBG |
| K272 | Glycyl lysine isopeptide | Uniprot |
| K277 | Ubiquitination | PSP |
| T293 | Phosphorylation | PSP |
| Y295 | Phosphorylation | PSP |
| K299 | Ubiquitination | PSP |
| K311 | N6-acetyllysine | Uniprot |
| K329 | Ubiquitination | PSP, TBG |
| K335 | Ubiquitination | PSP, TBG |
| K343 | Acetylation | PSP |
| K348 | Acetylation | PSP |

**Supplementary Table 3:** All reported post-translational modifications of human ATF4.
